# Nasopharyngeal gangrenous abscess with skull base extension caused by *Escherichia coli* after esophageal dilatation for esophageal reconstruction

**DOI:** 10.4103/2152-7806.69383

**Published:** 2010-09-16

**Authors:** Wing-Him Lau, Wei-Chieh Chang, Yuang-Seng Tsuei, Wen-Yu Cheng, Shao-Ching Chao, Chiung-Chyi Shen

**Affiliations:** 1 Department of Surgery, Cheng Ching General Hospital, Taichung, Taiwan; 2 Department of Neurosurgery, Taichung Veterans General Hospital, Taichung, Taiwan; 3 Faculty of Medicine, School of Medicine, National Yang-Ming University, Taipei, Taiwan

**Keywords:** Endonasal endoscopic, esophageal dilatation, esophageal stricture, esophageal reconstruction, nasopharyngeal abscess, *Escherichia coli*

## Abstract

**Background:**

Esophageal dilatation is the most widely used treatment option for the management of esophageal strictures. Complications include bleeding, brain abscess, esophageal perforation and bacteremia. Nasopharyngeal gangrenous abscess after the esophageal dilatation is very rare. Endonasal endoscopic surgery was performed to treat the lesion and a successful result was obtained.

**Case Description:**

A 59-year-old woman with a previous history of dilatation for esophageal stricture was admitted with a low-grade fever, headache, neck pain and cranial nerve abnormalities including sixth nerve palsy. Imaging studies aroused suspicion of necrotic retropharyngeal tumor with clivus, condylar process and cavernous sinus invasion. Biopsy with a pharyngosope was performed by an ENT doctor. The pathology showed acute necrotic inflammation, tissue granulation and bacteria colonies. Navigation with endonasal endoscopic surgery was chosen to treat the skull base and nasopharyngeal abscess. Bacterial culture showed *Escherichia coli*. Symptoms improved after the operation and treatment with antibiotics.

**Conclusion:**

A nasopharyngeal gangrenous abscess with extension to the skull base in the case of esophageal reconstruction after esophageal dilatation is extremely rare. Physicians dealing with esophageal stricture should keep in mind that a nasopharyngeal abscess is a potential complication of esophageal dilatation.

## INTRODUCTION

The most common procedure used for the esophageal stricture is esophageal dilatation which has dose associated risks. Esophageal perforation is the worst complication. The perforation rate is reported to range between 0.1 and 0.4%.[9] The rate of bacteremia after the procedure is high.[3] The infectious complications are rare.[2] But infections complications of the central nervous system following esophageal dilatation were seen in only a few reported cases.[1] Here, we presented a rare case of nasopharyngeal abscesses with extension to the clivus, left cavernous sinus and left parasellar region. The abscess was cultured by *Escherichia coli* after esophageal reconstruction with esophageal dilatation. The patient was successfully treated by a minimally invasive procedure with endonasal endoscopic approach to treat the abscess. The clinical course and the mechanisms of the abscess formation are discussed.

## CASE REPORT

A 59-year-old woman had a history of esophageal benign tumor after esophagectomy and reconstruction with gastric tube, 4 years ago. She had past history of neck pain radiating to the left shoulder and occipital region. Severe neck pain limited her range of motion. She had been admitted to a hospital with the suspicion of cervical disk herniation with cervical radiculopathy.

**Figure 1:**
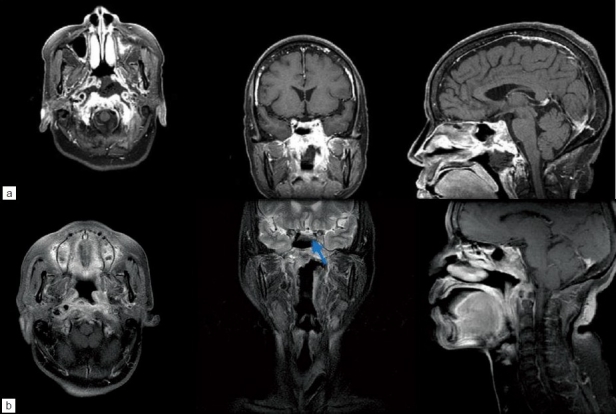
(a) MRI scan of sella. Preoperative axial, coronal and sagittal images after intravenous administration of gadolinium diethylenetriamine penta-acetic acid are shown. Thickening of the nasopharyngeal wall with irregular contrast enhancement is seen. Ill-defined enhancement over the skull base including the clivus, bilateral petrosal apex and condylar process of the occipital bone is also seen. Prominent enhancement over the left cavernous sinus and left parasellar region can also been seen. (b) Two months postoperative axial, coronal, and sagittal postgadolinium images show improvement. Residual left sixth nerve palsy was presented after the surgery, while other cranial neurolopathies improved. There were some residual abscesses in the left cavernous sinus and parasellar region (arrow)

Two months before admission, she complained of dysphagia, and esophageal dilatation was performed due to the stricture. One month later, she developed diplopia, slurred speech and dysphasia. Low-grade fever and whitish rhinorrhea were also noticed. On neurological examination, the patient was alert and oriented. She had severe neck pain, left sixth nerve palsy, mildly slurred speech and dysphagia. Other cranial nerves, including III, IV, V, had no defect. Muscle strength was normal in upper extremities. The deep-tendon reflexes were normal bilaterally. Sensory examination showed nothing unusual. Laboratory studies were within the normal range. A plain chest film showed a supradiaphragmatic gastric bulb. C-reactive protein levels were elevated to 3.6mg/dL (normal range 0.0-0.8 mg/dL). Magnetic resonance imaging (MRI) of sellar region disclosed a thickening of the nasopharyngeal wall with irregular contrast enhancement. Ill-defined enhancement was seen over the skull base including the left cavernous sinus, left parasellar region, clivus, bilateral petrosal apex, condylar process of the occipital bone and C1 vertebra [Figure 1a]. Nasopharynx carcinoma was first considered. A nasopharyngoscopy was performed by an ENT doctor and showed a necrotic lesion over the left nasopharynx. The pathological report of a punch biopsy showed acute necrotic inflammation, tissue granulation and bacteria colonies.

**Figure 2:**
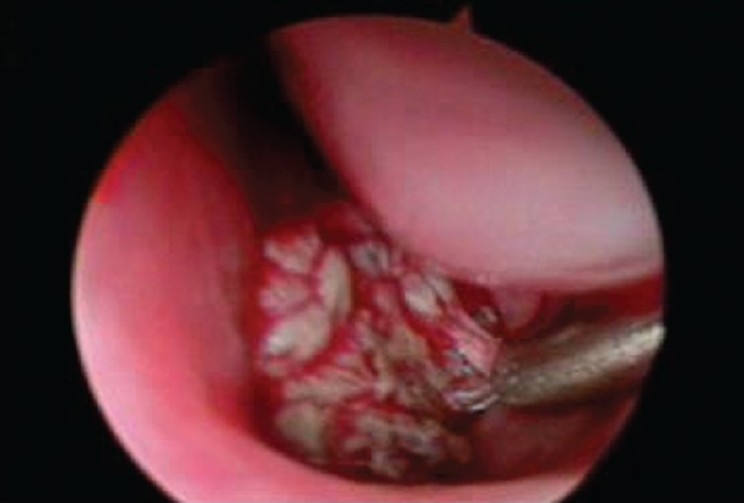
Operative finding under the endoscopy which showed the nasopharyngeal abscess

We took an endonasal endoscopic approach to treat the suprasellar and nasopharyngeal lesion. Gray brownish necrotic tissue of soft to firm nature was removed [Figure 2]. Pathology showed extensive acute gangrenous inflammation and sequestrum of bone. The culture from this specimen grew *E.coli*. Two weeks of Flomoxef therapy was given and the patient was discharged 22 days after operation and she presented with only mild left sixth nerve palsy. Two months later, MRI scan with DWI sequences showed residual abscesses in the left cavernous sinus and parasellar region [Figure 1b].

## DISCUSSION

Esophageal dilatation is the most widely used option for the treatment of esophageal strictures. It is associated with bacteremia in 22 -72% of the procedures[2,3] whereas, it occurs in 30-60% of the cases following dental extraction.[7] Microscopic perforation during dilatation is generally considered to be the site of bacterial entry.[11] Another hypothesis implicates the Batson’s venous plexus as the pathway of propagation of bacteria from the thorax to the brain.[12] The most commonly observed microorganism is *Streptococcus viridans*, probably originating as a contaminant from the oropharynx.[2] It is transient and not associated with clinical complications.[2] Central nervous system infections following esophageal dilatation have been reported.[12] However, a nasopharyngeal gangrenous abscess due to esophageal reconstruction following esophageal dilatation is extremely rare. Most of the reported complications of post-esophageal dilatation infections are spread to form supratentorial brain abscesses.[12] The case presented here is a rare case of nasopharyngeal gangrenous abscess with extension into the skull base, including cavernous sinus, parasellar region, clivus, bilateral petrosal apex and condylar process after esophageal dilatation in a case of esophageal reconstruction. Besides, the pathogen was *E. coli*, which is different from previous reports.

Retropharyngeal abscesses are uncommon but are potentially lethal. Abscesses in adults are usually secondary to the trauma of upper aerodigestive tract caused by foreign bodies or iatrogenic instrumentation such as dental procedures, feeding tube insertion, oral tracheal intubation, esophagoscopy, etc. The most commonly isolated pathogen is *Streptococcus pyogenes* followed by *Staphylococcus aureus*.[10] In literature, the *E. coli* infections have been reported only in a few cases.[5] None of them are followed by the esophageal dilatation. We believe that the pathological involvement of the *E. coli* infection in this case included esophageal stricture related to the supradiaphragmatic gastric bulb, esophageal dilatation complicated with retropharyngeal abscess and contiguous spread of inflammation to the cavernous sinus, parasellar region and clivus. Abducens palsy has been reported as a common complication of the clivus and paraclival pathology.[8]

A minimally invasive endonasal endoscopic surgery was commonly used for approached the abscesses over the clivus, sellar, parasellar region and cavernous sinus. The major advantage of this operation is that it provides the most direct anatomical route to the lesion without transversing the major neurovascular structures.[6] A stereotactic neuronavigation system is chosen to avoid further contamination of the central nervous system.

## CONCLUSION

A nasopharyngeal gangrenous abscess in the case of esophageal reconstruction with extension to the skull base following esophageal dilatation is extremely rare. Tissue sampling is important to differentiate an infectious disease from malignancy. In endonasal endoscopic surgery, the endoscope allows us an extended view over the skull base around the sellar region and onto the surrounding structures.[4] Physicians dealing with esophageal stricture should keep in mind that a nasopharyngeal abscess with skull base extension is a potential complication of esophageal dilatation.
